# Toward Ecologically Relevant Genetics of Interactions Between Host Plants and Plant Growth‐Promoting Bacteria

**DOI:** 10.1002/ggn2.202300210

**Published:** 2024-03-21

**Authors:** Rémi Duflos, Fabienne Vailleau, Fabrice Roux

**Affiliations:** ^1^ LIPME INRAE CNRS Université de Toulouse Castanet‐Tolosan 31326 France

**Keywords:** GWAS, microbiota, natural genetic variation, PGPB, SynCom

## Abstract

The social movement to reduce reliance on pesticides and synthesized fertilizers and the growing global demand for sustainable food supplies require the development of eco‐friendly and sustainable agricultural practices. In line, plant growth‐promoting bacteria (PGPB) can participate in creating innovative agroecological systems. While the effectiveness of PGPB is highly influenced by abiotic conditions and microbe–microbe interactions, beneficial plant–PGPB interactions can also highly depend on both host and PGPB genotype. Here, the state of the art on the extent of natural genetic variation of plant–PGPB interactions and the underlying genetic architecture, in particular in *Arabidopsis thaliana* is reviewed. Extensive natural plant genetic variation in response to PGPB is associated with a polygenic architecture and genetic pathways rarely mentioned as being involved in the response to PGPB. To date, natural genetic variation within PGPB is little explored, which may in turn allow the identification of new genetic pathways underlying benefits to plants. Accordingly, several avenues to better understand the genomic and molecular landscape of plant–PGPB interactions are introduced. Finally, the need for establishing thorough functional studies of candidate genes underlying Quantitative Trait Loci and estimating the extent of genotype‐by‐genotype‐by‐environment interactions within the context of realistic (agro‐)ecological conditions is advocated.

## Graphical abstract

In the context of current global changes reducing food security on a worldwide scale and causing substantial economic losses, harnessing natural genetic variation of interactions between plants and plant growth‐promoting bacteria represents an opportunity to identify new genetic and molecular pathways underlying beneficial plant–PGPB interactions, which will, in turn, participate in the development of eco‐friendly and sustainable agricultural practices.

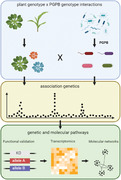

## 1 Introduction

Current global changes, including climate change (e.g., climate warming, flooding), habitat degradation, and long‐distance dispersal of pests due to global transport networks, result in yield losses in crop species, which in turn reduce food security on a worldwide scale and cause substantial economic losses.^[^ ^1^, ^2^, ^3^ ^]^ There is therefore an urgent need to develop innovative agro–ecological strategies that will address both the growing global demand for sustainable food supplies and the social movement to reduce reliance on pesticides and synthesized fertilizers. While strategies such as crop/varietal mixtures, temporal crop diversification, and plant breeding for alleviating abiotic and biotic stresses have been successfully developed and implemented to increase and stabilize crop yields across diverse spatiotemporal scales,^[^ ^4^, ^5^, ^6^, ^7^ ^]^ the exploration and leverage of plant microbiota represent an additional opportunity to participate in the development of eco‐friendly and sustainable agricultural practices.^[^ ^8^ ^]^

Often referred to as the second genome,^[^ ^9^ ^]^ plant microbiota are sets of microbes of a particular compartment, such as roots, leaves, stems, flowers or seeds.^[^ ^10^ ^]^ Plant microbiota includes, among others, plant growth‐promoting bacteria (PGPB) that correspond to bacterial strains isolated from diverse environmental reservoirs with the potential to provide diverse benefits to wild plants and crops.^[^ ^11^ ^]^ For instance, PGPB can mobilize and provide nutrients to plants and alleviate abiotic stresses such as drought and salinity.^[^ ^12^, ^13^ ^]^ PGPB can also provide protection against pathogens and pests, either directly (e.g., production of antimicrobial components) or indirectly (elicitation of plant defense).^[^ ^14^, ^15^ ^]^ PGPB represent therefore a unique opportunity to develop sustainable and eco‐friendly agricultural practices, with the use of PGPB as biostimulants, biofertilizers, or biocontrol agents.^[^ ^16^, ^17^ ^]^

The combination of diverse “omics” tools (including comparative genomics, metagenomics, transcriptomics and proteomics) with mutational studies revealed a large number of genes and genetic pathways involved in key steps of endophytic colonization by PGPB (i.e., recognition of root exudates and motility toward the plant, adhesion to the surface of roots and leaves, biofilm formation, plant surface penetration and colonization of the internal parts of a plant) or underlying the benefits conferred by PGPB to plants.^[^ ^8^, ^18^ ^]^ For instance, in phylogenetically distant PGPB, the pyrroloquinoline quinone‐encoding genes *pqqBCDEFG* can promote plant growth by improving plant nutrition through mineral phosphate solubilization.^[^ ^19^ ^]^ The production of siderophores by PGPB can stimulate plant growth by providing iron, an essential nutrient for a variety of cellular functions in plants.^[^ ^20^, ^21^ ^]^ In addition, the negative effects of abiotic stresses on plant growth can be alleviated by the activity of the 1‐aminocyclopropane‐1‐carboxylate (ACC) deaminase gene *acdS*, which degrades a plant ethylene precursor that accumulates under stress conditions and can cause cell necrosis.^[^ ^22^ ^]^ Finally, PGPB may have biocontrol properties linked to gene clusters encoding compounds with antibacterial and antifungal activities^[^ ^18^ ^]^ or to the priming of plant systemic immunity, with a faster and stronger activation of plant defense responses upon pathogen attack.^[^ ^23^ ^]^ A combination of “omics” tools with mutational studies was also adopted to reveal key plant genetic features involved in the dialog of the plants with PGPB,^[^ ^8^ ^]^ such as plant receptors,^[^ ^24^ ^]^ hormone signaling pathways (notably jasmonic acid, ethylene, and salicylic acid),^[^ ^25^ ^]^ small RNAs^[^ ^26^ ^]^ and cell growth.^[^ ^8^ ^]^ Interestingly, by challenging the model plant *Arabidopsis thaliana* to 39 endophytic bacterial strains, a transcriptomic study revealed a molecular response, termed the general non‐self response (GNSR) that involves the expression of a core set of 24 genes, being significantly associated with mechanisms involved in plant immunity.^[^ ^27^ ^]^

While combining “omics” tools with the use of artificial genetic variation was highly informative, another strategy for identifying the genes and genetic pathways involved in the dialog between plants and PGPB is to leverage natural genetic variation segregating within both plant species and PGPB species. However, exploring the natural genetic variation of plant–PGPB interactions has been overlooked. Yet, in plant pathosystems, harnessing natural genetic variation has revealed a wide range of molecular functions, which have rarely been considered to be associated with plant immunity when based on artificial genetic variation.^[^ ^6^, ^28^, ^29^, ^30^ ^]^ For instance, a genome‐wide association study (GWAS) followed by the complementation of a mutant line with two natural alleles identified the atypical kinase RKS1 as underlying a major Quantitative Trait Loci (QTL) of response to the bacterial pathogen *Xanthomonas campestris* pv. *campestris*.^[^ ^31^ ^]^ Then, the combination of a transcriptomic study performed on transgenic lines deregulated for the expression of *RKS1* (i.e., a knock‐out (KO) mutant line, two amiRNA lines, and two lines overexpressing *RKS1*) with a systems biology based network reconstruction, led to the reconstruction of *RKS1*‐dependent signaling pathways, which were largely distinct from PAMP‐triggered immunity (PTI) and effector‐triggered‐immunity (ETI).^[^ ^32^ ^]^

Here, we review the state of the art on the extent of natural genetic variation of plant–PGPB interactions at the intraspecific level and the underlying genetic architecture. We first review the extent of natural genetic variation of plants in response to PGPB and highlight the main conclusions of the few GWAS conducted on the plant side. We then review the extent of natural genetic variation within PGPB species for their benefits conferred to plants and note the absence of a large collection of natural strains of a given PGPB that would allow setting up GWAS. We finally introduce several avenues that need to be explored in the future to obtain a thorough understanding of the genetic and molecular bases underlying natural variation of plant–PGPB interactions within the context of realistic (agro‐)ecological conditions.

## 2 Extensive Natural Plant Genetic Variation in Response to PGPB is Associated with a Polygenic Architecture and Genetic Pathways Rarely Mentioned in the Literature as Involved in the Response to PGPB

**Figure 1:**
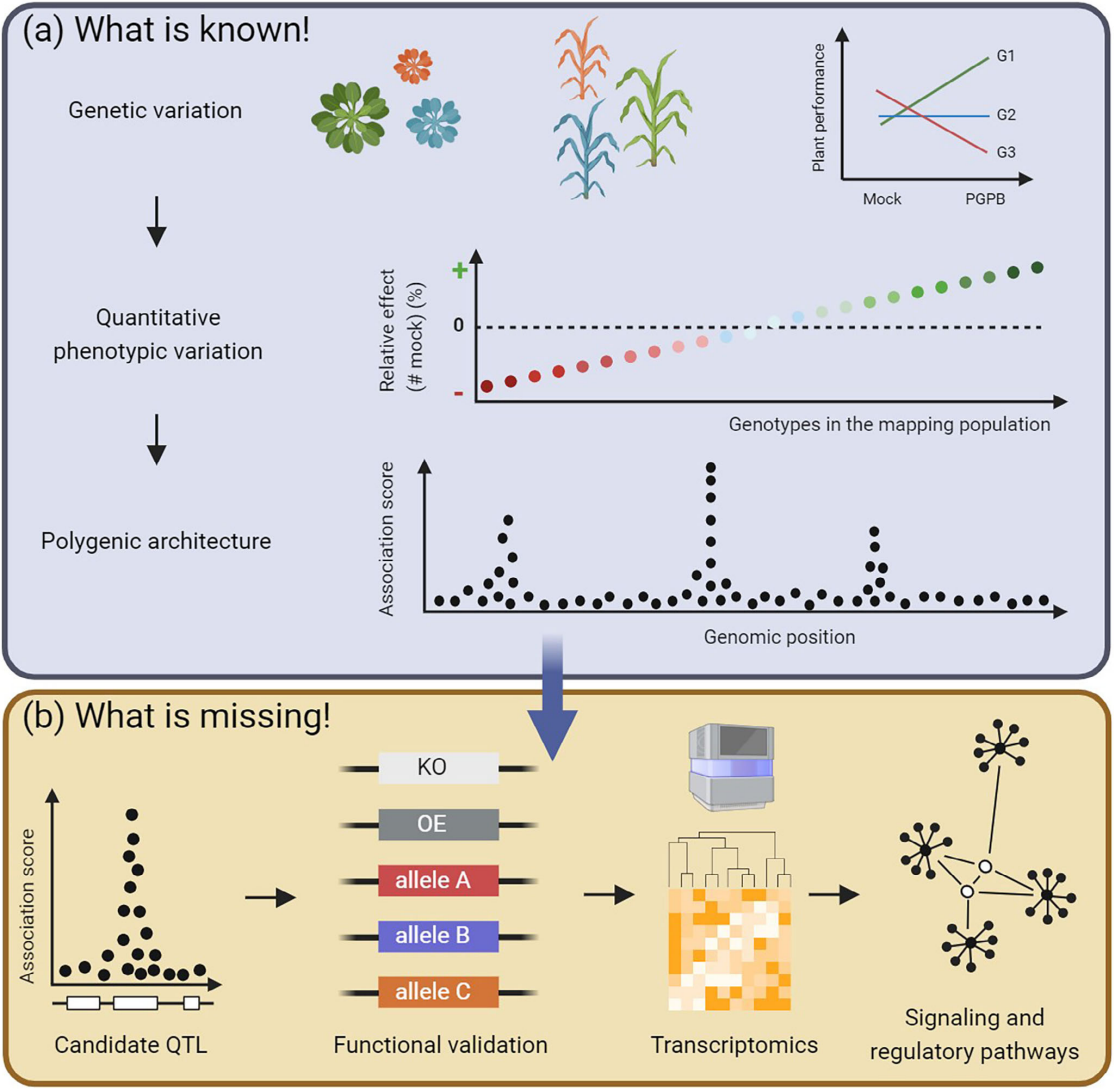
State of the art of the genetics of natural genetic variation of plant response to PGPB. a) Illustration of what is known about the extent of plant genetic variation in response to PGPB and the underlying genetic architecture. The interaction plot on top right illustrates the genetic variation between three plant genotypes after inoculation with a PGPB. The green, blue, and red lines correspond to a beneficial, neutral, and negative bacterial effect on genotypes G1, G2, and G3, respectively. Quantitative phenotypic variation in response to a PGPB ranging from negative to beneficial effects is represented by a gradient from red to green. Manhattan plot illustrating of polygenic architecture after performing a GWA mapping that enables a fine‐mapping of QTLs. b) Illustration of the next steps for a better understanding of the genetic and molecular mechanisms of the natural variation of plant response to PGPB. KO: knock‐out mutant line. OE: overexpressor line. Illustration of transcriptomics studies performed with an Illu**m**ina NextSeq 2000 sequencer. See text for details. Created with BioRender.com.

As a first step to investigate the extent of natural plant genetic variation in response to PGPB, most studies challenged a very low number of genotypes (typically less than 10 genotypes) with either a single PGPB strain^[^ ^33^, ^34^, ^35^ ^]^ or a consortium of PGPB strains.^[^ ^36^, ^37^ ^]^ These studies tested an effect of PGPB mainly on plant growth,^[^ ^34^ ^]^ to a lesser extent on yield production^[^ ^38^, ^39^ ^]^ and as biocontrol agents against bacterial,^[^ ^33^, ^37^ ^]^ fungal^[^ ^35^ ^]^ and oomycete^[^ ^40^ ^]^ pathogens. These studies were conducted on several genotypes of either wild plants such as *A. thaliana* ^[^ ^37^, ^41^ ^]^ or crops such as *Zea mays* ^[^ ^38^ ^]^ and *Oryza sativa*.^[^ ^42^ ^]^ Plants were inoculated with one or several potential PGPB strains isolated either from the native plants,^[^ ^37^, ^41^ ^]^ non‐native plants,^[^ ^38^ ^]^ or with commercial strains.^[^ ^43^ ^]^ Despite the very low number of plant genotypes challenged with PGPB, most studies indicate that the beneficial effect conferred by a specific PGPB was highly dependent on the host genotype (**Figure** **1**a), albeit specific to the phenotypic trait measured. For instance, a significant genetic variation was detected between two genotypes of *O. sativa* in response to *Burkholderia kururiensis* M130 for bacterial root colonization but not for the number of lateral roots.^[^ ^44^ ^]^ Similarly, a significant genetic variation was detected among 16 genotypes of *Z. mays* in response to *Herbaspirillum seropedicae* for root‐related traits but not for shoot‐related traits.^[^ ^45^ ^]^

The full extent of plant genetic variation in response to PGPB was estimated in eight GWAS with the phenotyping of the effect of PGPB on a substantial number of genotypes (mean ≈260, min = 118, max = 360) (Table S1, Supporting Information). While four GWAS were conducted on *A. thaliana*,^[^ ^46^, ^47^, ^48^, ^49^ ^]^ the other four GWAS were conducted on two crops, *Z. mays* (*n* = 3)^[^ ^50^, ^51^, ^52^ ^]^ and *Triticum aestivum* (*n* = 1)^[^ ^53^ ^]^ (Table S1, Supporting Information). Plants were inoculated with either one bacterial strain (*Azoarcus olearius* DQS‐4^T^, *Azospirillum baldaniorium* Sp245, *A. brasilense* Ab‐V5, *Bacillus pumilus* TUAT‐1, *Pseudomonas simiae* WCS417r, *Pseudomonas siliginis* OTU6_Psi_1) in six GWAS or a consortium of four bacterial strains (*A. brasilense* Ab‐V5, *Bacillus thuringiensis* RZ2MS9, *Delftia* sp. RZ4MS18, *Pantoea agglomerans* 33.1) in the two other GWAS (Table S1, Supporting Information).^[^ ^51^, ^52^ ^]^ Most phenotypic traits measured in these GWAS were related to plant growth, in particular traits related to root architecture (Table S1, Supporting Information). To our knowledge, no GWAS conducted on plants was based on the phenotyping of the effect of PGPB on yield (e.g., seed production) or a biocontrol effect by a PGPB. Extensive plant genetic quantitative variation in response to PGPB was detected in each GWAS (Figure 1a), with heritability estimates reaching up to 0.81.^[^ ^49^ ^]^ Heritability estimates were nonetheless highly dependent on the measured trait and the growth conditions (Table S1, Supporting Information). Interestingly, each GWAS revealed contrasted responses among host genotypes to PGPB inoculation, ranging from beneficial to negative responses (Figure 1a; Table S1, Supporting Information), thereby suggesting that declaring a strain or a consortium of strains as PGPB might be highly dependent on the host genotype tested. Each GWAS revealed a highly polygenic architecture of the plant response to PGPB (Figure 1a; Table S1, Supporting Information). In addition, significant signatures of local adaptation were detected on the candidate genes underlying the QTLs of *A. thaliana* detected in response to *P. siliginis*,^[^ ^49^ ^]^ thereby suggesting co‐evolutionary trajectories in this native host‐PGPB interaction. The enriched pathways associated with the list of candidate genes largely vary among the GWAS, which is in line with the complexity of the interplays between host plants and PGPB highlighted by “omics” and mutational studies.^[^ ^8^, ^18^ ^]^ Nonetheless, despite the low number of GWAS, some enriched pathways were found in two or more GWAS, such as pathways involved in primary and secondary metabolism and transport (Table S1, Supporting Information).

The next step to better understand the genetic and molecular mechanisms of the natural variation of plant response to PGPB relies on the functional validation of candidate genes underlying detected QTLs (Figure 1b). For instance, GWAS on the plant response to pathogens were followed by the functional validation of 59 QTLs, which in turn allowed the identification of new genes and molecular functions involved in plant immunity.^[^ ^6^ ^]^ In contrast, to our knowledge, no QTL associated with plant response to PGPB has been functionally validated yet. Although the traditional approach for functional validation involves the phenotyping of lines deregulated for the expression of the candidate genes, such as KO mutant lines and overexpressor (OE) lines,^[^ ^31^, ^32^ ^]^ we stress the need to also functionally validate candidate genes by complementation of a KO mutant line with natural alleles (Figure 1b). Indeed, the allelic effect observed between two natural alleles may not reflect the phenotypic differences observed between a wild‐type genotype and a mutant line, as previously observed for diverse phenotypic traits such as flowering time^[^ ^54^ ^]^ and pathogen resistance.^[^ ^30^, ^55^, ^56^ ^]^ The generation of these isogenic lines can be then used for addressing changes in global gene expression linked to the candidate genes (Figure 1b). Such transcriptomics studies combined with network reconstruction and mutational analysis can in turn help to identify and validate molecular networks, in particular signaling and regulatory pathways of the response to PGPB (Figure 1b). This integrative approach has proved to be successful in identifying new pathways in plant immunity.^[^ ^32^ ^]^ Because molecular networks might highly depend on the plant–PGPB combination, a comparative network analysis based on systems biology may help to identify common signaling and regulatory pathways involved in the plant response to PGPB.^[^ ^5^ ^]^

We should however stress that while the creation of KO or OE lines is feasible in established genetic systems such as *A. thaliana* and few major crops (e.g., maize, rice, and soybean), this remains a challenge for the large majority of crops. For those crops, an approach combining transcriptomics studies and network reconstruction can nevertheless be applied on near‐isogenic lines or heterogeneous inbred families.^[^ ^57^ ^]^

## 3 Exploring the Natural Genetic Variation of PGPB to Identify New Genetic Pathways Underlying their Potential Benefits on Plants

The identification of natural strains with a potential beneficial effect on plant growth,^[^ ^34^ ^]^ abiotic stress alleviation,^[^ ^58^ ^]^ and plant health^[^ ^35^, ^59^ ^]^ was mainly based on screening a large number of strains. These strains were mainly isolated from the root and leaf compartments^[^ ^42^, ^60^, ^61^, ^62^, ^63^ ^]^ or, to a lesser extent, from the rhizosphere.^[^ ^64^ ^]^ While successful, most of these studies focused on genetic variation among potential PGPB species. The small number of studies focusing on exploring genetic variation within potential PGPB species may originate from the extensive use of evolutionary conserved genes that are hypervariable but have a low taxonomic resolution, such as the 16S rRNA gene,^[^ ^65^ ^]^ which additionally can be present in multiple copies in a large fraction of bacterial species.^[^ ^66^ ^]^ Recently, amplicon amplification of genes with a deeper taxonomic resolution and present in a single copy in most bacterial genomes has facilitated the isolation of multiple strains of a given PGPB species. For instance, combining the use of selective media and amplification of an amplicon of the *gyrB* gene, which encodes for the bacterial gyrase *β*‐subunit, allowed the isolation of six strains of *P. siliginis*, a bacterial species with a PGPB effect on *A. thaliana*.^[^ ^41^, ^49^ ^]^ Similarly, amplification of an amplicon of the *rpoD* gene, which encodes an RNA polymerase primary σ^70^, allowed the identification of two PGPB strains of *Pseudomonas mediterranea*, both with a biocontrol activity against the fungal pathogens *Fusarium oxysporum*, *Fusarium moniliforme* and *Pyricularia oryzae*.^[^ ^67^ ^]^ However, we must emphasize the importance of sequencing the genomes of bacterial strains to validate their taxonomic affiliation. For instance, amplification of 16S rRNA and *rpoD* amplicons on 137 bacterial strains isolated from the rhizosphere and leaf compartment of *Solanum tuberosum* first led to the identification of nine phylogenetically close strains of the genus *Pseudomonas*, with biocontrol effects against the oomycete pathogen *Phytophthora infestans*.^[^ ^68^ ^]^ Genome sequencing further revealed that these nine strains were affiliated to either *Pseudomonas fluorescens*, *Pseudomonas koorensis* or *Pseudomonas chlororaphis*.^[^ ^69^ ^]^ Similarly, while the third most abundant operational taxonomic unit (OTU) in the leaf and root microbiota of 163 natural populations of *A. thaliana* located in the south–west of France was first affiliated to the genus *Collimonas* based on *gyrB* amplification,^[^ ^70^ ^]^ genome sequencing of four strains from this OTU with a PGPB effect led to the identification of two new species belonging to the *Oxalobacteraceae* family.^[^ ^41^ ^]^

Comparative genomics has been used at the interspecific level in several studies to identify genes underlying natural genetic variation of PGPB effects. For instance, based on the accessory genomes of nine whole‐genome sequenced *Pseudomonas* sp. strains, 65 genes were positively correlated but not functionally validated, with an anti‐oomycete activity in an in vitro assay.^[^ ^69^ ^]^ Another study was recently conducted at the interspecific level on 99 commensal strains isolated from *A. thaliana* and clustered by 99% 16S rRNA gene homology in 10 OTUs of the genus *Pseudomonas*.^[^ ^71^ ^]^ These 99 strains were tested *in planta* for their biocontrol potential against a strain of the bacterial pathogen *Pseudomonas viridiflava* inoculated on *A. thaliana*. Based on a gene presence/absence matrix, comparative genomics performed at the intra‐OTU (OTU2, OTU3 and OTU4) and inter‐OTU levels revealed 14 genes associated with plant protection against the pathogenic strain, nine genes being specific to OTU2.^[^ ^71^ ^]^ The co‐inoculation of the pathogenic strain with eight mutants derived from the protective OTU2, suggests that candidate genes related to iron uptake and biofilm formation participate in the protection of *A. thaliana* against *P. viridiflava*.^[^ ^71^ ^]^

By contrast, to our knowledge, no GWAS has been performed on the effects of a specific PGPB species on plants. GWA mapping within a PGPB species should nevertheless be achievable. First, substantial genetic variation for a beneficial effect on plants has been observed among strains of a given PGPB species, as previously observed with *A. brasilense* on *T. aestivum* root growth,^[^ ^39^ ^]^ *Pseudomonas aeruginosa* on the control of the oomycete pathogen *Phytophthora meadii*,^[^ ^61^ ^]^ and diverse PGPB species (e.g., *Paraburkholderia fungorum*, *Pseudomonas moraviensis* and *P. siliginis*) on the vegetative growth of *A. thaliana*.^[^ ^41^ ^]^ Second, GWA mapping has been proven successful in fine mapping of QTLs associated with pathogen virulence in the bacteria‐based pathosystems *A. thaliana* ‐ *Xanthomonas arboricola*,^[^ ^72^ ^]^ *O. sativa* ‐ *Xanthomonas oryzae* pv. *oryzae* ^[^ ^73^ ^]^ and *Capsicum annuum ‐ Xanthomonas euvesicatoria*.^[^ ^74^ ^]^

**Figure 2:**
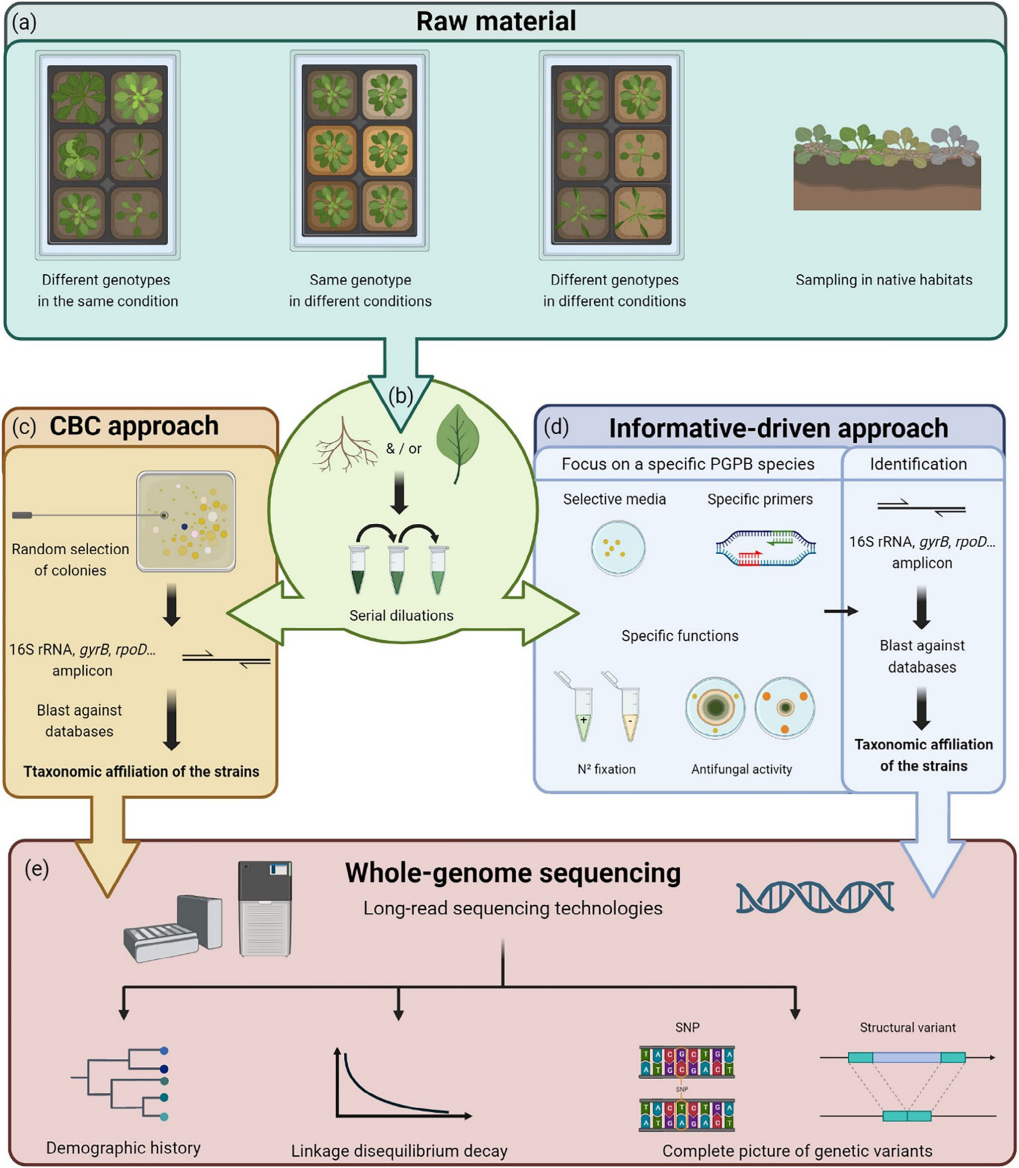
Steps for conducting a successful GWAS in a PGPB species. a) Sampling strategy to isolate genetically distinct strains of a specific PGPB species. b) Sampling root and/or leaf tissues for plating serial dilutions on media after grinding. Isolation of tens of strains of a specific PGPB species can be achieved by a community‐based culture approach c) and/or an informative‐driven approach d). e) Sequencing strategy for an accurate taxonomic affiliation and obtaining key information prior to GWA mapping. Illustration of long‐read sequencing with Nanopore PromethION 48 and a PacBio Sequel. The structural variant corresponds to a gene deletion. Created with BioRender.com.

Successfully conducting a GWAS in a given PGPB species requires the creation of a large collection of genetically distinct strains (**Figure** **2**). To do so, because plant microbiota can highly depend on both (native or controlled) growing conditions and plant genotype,^[^ ^10^, ^70^, ^75^, ^76^ ^]^ we strongly recommend a sampling of leaf and root tissues from different genotypes grown on diverse soils, or from different plants from a large range of native habitats (Figures 2a,b). In the latter case, an ecological characterization of the native habitats, such as climate variables, might allow defining the ecological niches of PGPB^[^ ^77^ ^]^ and performing Genome‐Environment Association (GEA) analyses.^[^ ^57^, ^78^, ^79^ ^]^ To our knowledge, no GEA analysis was performed to identify the adaptive genes associated with climate variation. Yet, the identification of such genes is of primary importance if one wants to develop and apply biostimulants, biofertilizers or biocontrol agents on crops that can be efficient in a large range of climate conditions. This is particularly relevant in the context of climate change.

The establishment of the collection can result from the complementarity of two approaches, namely i) a community‐based culture (CBC) approach based on a random picking of colonies on one or several media (Figure 2c),^[^ ^80^ ^]^ and ii) an informative‐driven approach based on the use of selective media, the use of specific primers and the search for specific functions such as nitrogen fixation, biosynthesis of indole‐3‐acetic acid (IAA) or antifungal activities (Figure 2d). Followed by the sequencing of a housekeeping gene amplicon for taxonomic affiliation,^[^ ^29^ ^]^ combining these two approaches allowed the isolation of several strains of seven common PGPB species of the *A. thaliana* leaf microbiota.^[^ ^41^ ^]^

To successfully conduct a GWAS in a given PGPB species, we advocate for the whole‐genome sequencing of the isolated strains with long‐read sequencing technologies (Figure 2e). As previously mentioned, whole‐genome sequences will allow for a better taxonomic affiliation of the strains using for instance FastANI, an algorithm representing the Average Nucleotide Identity (ANI) of all orthologous genes shared between two genomes.^[^ ^81^ ^]^ They will also provide key information before running GWA mapping, such as i) the potential effects of population structure, related to the demographic history of the strains, on inflating the rate of false–positive associations and ii) an estimate of the linkage disequilibrium (LD) decay (Figure 2e).^[^ ^6^ ^]^ Although the LD extent is assumed to be long in asexually reproducing bacteria, a relatively short LD extent (<100 bp) was detected in the bacterial pathogen *X. arboricola*, thereby allowing the fine mapping of candidate genes critical for pathogenicity.^[^ ^72^ ^]^ Finally, whole‐genome sequences will provide a complete picture of genetic variants, including structural variants (e.g., presence/absence of genes) (Figure 2e) that can be used by cutting‐edge statistical models used in GWA mapping on human bacterial pathogens.^[^ ^82^ ^]^

## 4 Future Prospects

Here, we introduce several avenues to be explored in the future to obtain a thorough understanding of the genetic and molecular bases underlying natural genetic variation of the plant–PGPB interactions in a context of realistic (agro‐)ecological conditions.

### 4.1 GWAS in Plants: Make the Way to Synthetic Communities!

**Figure 3:**
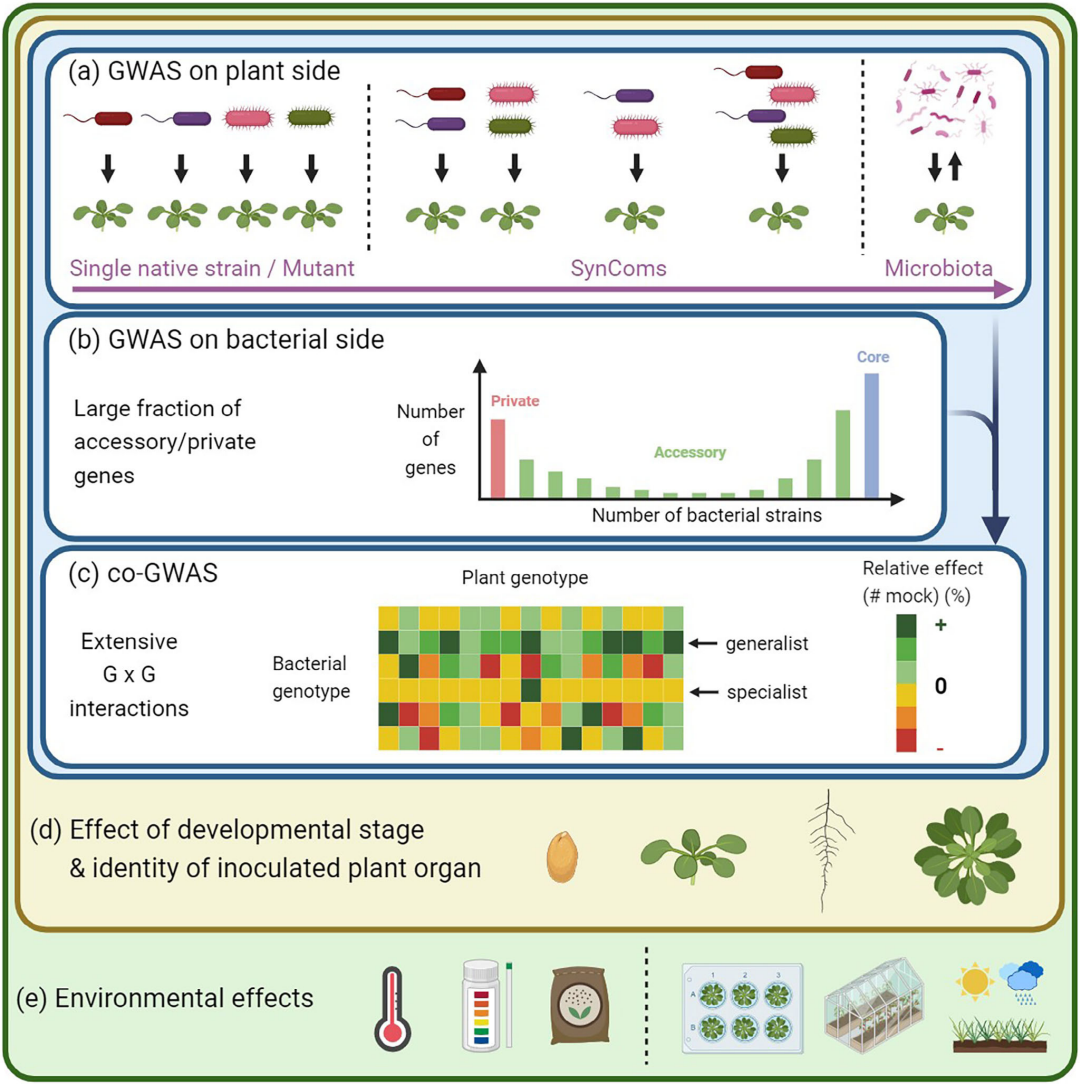
Future avenues on the ecological genetics underlying natural variation of plant–PGPB interactions. a) GWAS in plants. From mono‐inoculation to microbiota: toward inoculating plants with SynComs. b) GWAS in PGPB. Considering the large fraction of private and accessory genes to run GWA mapping. c) Matrix illustrating genotype‐by‐genotype interactions between a host plant and a PGPB species. d) Effects of the inoculated plant organ and development stage on the genetics of plant–PGPB interactions. e) Effects of environmental gradients on the genetics of plant–PGPB interactions. Created with BioRender.com.

As previously mentioned, most GWAS were conducted on plants inoculated with one natural bacterial strain (Table S1, Supporting Information). Interestingly, a recent GWAS on the response of *A. thaliana* to four type III effector mutants of *Ralstonia solanacearum*, one of the most devastating bacterial pathogens worldwide, led to the identification of a susceptible *NLR* gene mediating a negative regulation of the host plant immune response.^[^ ^30^ ^]^ This *NLR* gene has not previously been found in GWAS conducted on plants inoculated with the GMI1000 wild‐type strain from which the four mutants are derived.^[^ ^30^, ^56^, ^83^ ^]^ It might therefore be of interest to conduct GWAS on plants in response to PGPB strains that are mutated for key genes involved in endophytic colonization or conferring benefits to the plants (**Figure** **3**a).

On the opposite side of inoculating plants with a single PGPB strain, several studies in association genetics revealed the polygenic architecture of plants interacting with natural bacterial communities,^[^ ^84^, ^85^, ^86^, ^87^, ^88^, ^89^, ^90^, ^91^, ^92^ ^]^ which may include a large number of PGPB species,^[^ ^41^ ^]^ in field conditions or native habitats. An intermediate situation between inoculation with a single PGPB strain and studying the genetics of host plants interacting with its natural bacterial microbiota would be to inoculate plants with synthetic communities (SynComs) of PGPB, as performed on *Z. mays* with 360 inbred lines inoculated with a SynCom of four PGPB (Figure 3a).^[^ ^51^, ^52^ ^]^ While informative, this study did not include the individual inoculation of the 360 inbred lines with each member of the SynCom, thereby precluding testing whether the genetic architecture of response to co‐inoculation with several PGPB species/strains can be predicted from the genetic architecture of response to mono‐inoculation with each member of the SynCom. For instance, in the context of plant–plant interactions, the identification of QTLs of the response of a focal plant species to complex plurispecific mixtures of neighboring plants cannot be predicted from the identification of QTLs of the response of the same focal species to bispecific interactions with these neighboring plants.^[^ ^93^, ^94^ ^]^

### 4.2 GWAS Within a PGPB: Including the Accessory Genome to Conduct Successful GWAS

Similar to bacterial pathogens, sequencing the genome of several strains of the same PGPB species revealed a non‐negligible fraction of genes that were not common among strains (Figure 3b). For instance, using the PacBio long‐read sequencing technology, the genome sequencing of only six *P. siliginis* strains isolated from the leaf compartment of *A. thaliana* indicated that 4.4% and 12.5% of the 5185 identified orthogroups correspond to the private (i.e., orthogroups present in only one strain) and accessory (i.e., orthogroups present between two and five strains) genomes, respectively.^[^ ^41^ ^]^ Referred to as the dispensable genome, the private and accessory genomes can originate from intra‐genomic processes such as gene duplication followed by non‐functionalization, neofunctionalization or subfunctionalization^[^ ^95^, ^96^ ^]^ or from Horizontal Gene Transfer among strains of the same or different bacterial species.^[^ ^97^, ^98^ ^]^ Because both intra‐ and inter‐genomic processes may lead to the acquisition of novel adaptive traits,^[^ ^99^, ^100^ ^]^ there is an urgent need to develop new GWA mapping methodologies that include simultaneously, and not separately, the core and accessory genomes.^[^ ^82^ ^]^

### 4.3 Toward Co‐GWAS: Detection of Genotype‐by‐Genotype Interactions

The inoculation of three strains of *A. brasilense* on five genotypes of *T. aestivum* revealed that the outcome of plant–PGPB interactions (especially for root‐related traits) was highly dependent on both plant genotype and PGPB genotype.^[^ ^39^ ^]^ A similar conclusion was drawn from a study in which eight *A. thaliana* accessions from the south–west of France were inoculated with at least two strains of six PGPB species isolated from the leaf microbiota of *A. thaliana* and originating from the same geographic area.^[^ ^41^ ^]^ The detection of genotype‐by‐genotype (G × G) interactions in plant–PGPB systems^[^ ^101^ ^]^ is reminiscent of previous studies conducted in diverse bacteria‐based pathosystems.^[^ ^70^, ^72^, ^73^, ^102^ ^]^

The presence of G × G interactions is a prerequisite for studying co‐evolutionary processes and conducting co‐GWAS (Figure 3c).^[^ ^29^, ^103^, ^104^ ^]^ Joint‐GWA mapping can be performed on an extensive phenotypic dataset designed to estimate the joint genetic effects of the plant and the PGPB species, which can in turn allow the characterization of the underlying genetic architecture of G × G interactions, such as the number of inter‐genomic epistatic QTLs, their physical locations in their respective genomes and their corresponding allelic effects. Co‐evolution signatures can be then inferred on the detected QTLs.^[^ ^104^ ^]^ Because obtaining a large phenotypic dataset might be time‐consuming and require the development of automated high‐throughput phenotyping platforms adapted to plant–PGPB interactions (in particular at the root level), another approach to describe the genomic landscape of plant–PGPB interactions is to conduct free‐phenotyping co‐GWAS,^[^ ^29^ ^]^ also called natural co‐GWAS.^[^ ^103^, ^104^ ^]^ Successfully applied in several human–pathogen systems^[^ ^105^, ^106^, ^107^, ^108^ ^]^ and at least one animal–pathogen system,^[^ ^109^ ^]^ natural co‐GWAS jointly associate genome‐wide polymorphisms of hosts with genome‐wide polymorphism data of microbes (either harmful or beneficial) obtained from natural populations where both hosts and microbes cooccur.^[^ ^29^ ^]^ Combining paired plant and PGPB genomic information represents therefore an exciting opportunity to describe the adaptive genetic and molecular dialog underlying plant–PGPB interactions.

### 4.4 Considering the Effects of Inoculated Plant Organ and Environmental Gradients on the Genetics of Plant–PGPB Interactions

The respective contribution of natural intraspecific genetic variation of both plant and PGPB to plant health and productivity, as well as the heritability of plant × PGPB interactions, can strongly depend on the identity of the plant organ inoculated, as well as the plant developmental stage of PGPB inoculation (Figure 3d).^[^ ^110^ ^]^ For instance, in a study with eight *A. thaliana* accessions inoculated with at least two strains of six PGPB species, the extent of plant genetic variation, PGPB genetic variation, and G × G interactions were remarkably dependent on whether PGPB strains were inoculated on seeds or at the seedling stage.^[^ ^41^ ^]^

In addition, because climatic conditions, soil physico‐chemical properties, and microbe–microbe interactions can deeply affect the effectiveness of PGPB,^[^ ^111^, ^112^, ^113^, ^114^ ^]^ it would be necessary to examine the effect of abiotic and biotic stresses on the genetic and molecular mechanisms underlying natural genetic variation of plant–PGPB interactions (Figure 3e). For instance, under greenhouse conditions, the growth of *Avena sativa* was strongly affected by the interaction effects between 10 *A. sativa* cultivars, 16 PGPB species, and the level of fertilization.^[^ ^34^ ^]^ While informative, the extent of genotype‐by‐genotype‐by‐environment (G × G × E) interactions remains to be estimated using several strains of the same PGPB species and diverse abiotic or biotic stresses, tested separately or in combination.^[^ ^3^ ^]^

## 5 Conclusion

Besides the effect of climatic conditions, soil agronomic properties, and microbe–microbe interactions on the effectiveness of PGPB,^[^ ^111^, ^112^ ^]^ our review highlighted that beneficial interactions between plants and PGPB can highly depend on the genotype of both host plant and PGPB.^[^ ^41^, ^101^ ^]^ There is therefore a need for a better understanding of the genetic and molecular bases underlying natural genetic variation of plant–PGPB interactions, with the potential of discovering new genetic pathways underlying beneficial plant–PGPB interactions, notably on the bacterial side. However, we should stress that GWAS on plant response to PGPB were mainly conducted on genetic model systems, in particular *A. thaliana*. We therefore call for conducting GWAS on a large number of crop species, as previously done for disease resistance with GWAS conducted on 29 crop species.^[^ ^6^ ^]^ To do so would help to establish comparative GWAS that might help to identify common pathways involved in the response to PGPB.

Because finding the most efficient eco‐friendly solutions is a lofty goal in an ever more human‐driven multi‐stress environment, estimating the relative fraction of variance of beneficial plant–PGPB interactions explained by G × G × E interactions for diverse abiotic and biotic factors as well as growth conditions (e.g., agricultural management practices) appears as an essential prerequisite to develop personalized agricultural practices, which will in turn satisfy the growing global demand for sustainable food supplies and the need to reduce the use of synthesized fertilizers and pesticides.

## Conflict of Interest

The authors declare no conflict of interest.

## Supporting information

Supplemental Table 1
